# Supplementary material for: ‘Mystery big cats’ in the Peruvian Amazon: morphometrics solve a cryptozoological mystery
Source: PeerJ. 2014 Mar 6;2:e291. doi: 10.7717/peerj.291 (PMC3961146; doi:10.7717/peerj.291)
Supplement: Supplemental Information 1 [file peerj-02-291-s001.doc]

**‘Mystery big cats’ in the Peruvian Amazon: morphometrics solve a cryptozoological mystery**

**Darren Naish**^1*^**, Manabu Sakamoto**^2^**, Peter Hocking**^3^**, Gustavo Sanchez**^4^

^1^ *Ocean and Earth Science, National Oceanography Centre, Southampton, University of Southampton, Southampton SO14 3ZH, UK*

^2^ *School of Earth Sciences, University of Bristol, Bristol, BS8 1RJ, UK*

^3^ *Natural History Museum of the University of San Marcus, Lima, Peru*

^4^ *Fundación Neotrópico, La Laguna, 38208, Tenerife, Canary Islands, Spain*

* *Author and address for correspondence (*[*eotyrannus@gmail.com*](mailto:eotyrannus@gmail.com)*)*

**Data on whereabouts of specimens**

The original specimens are retained in the collections of the Natural History Museum of the National Higher University of San Marcos, Lima, Peru, where they are accessioned as MHN 9397 (‘anomalous jaguar’) and MHN 8736 (‘Peruvian tiger’). High quality casts were created by making plaster molds, then by making acrylic casts from the molds. It is these replicas that were used for our study. The casts are housed at the Fundación Neotrópico, La Laguna, Tenerife, Canary Islands where they are accessioned as CF-0022 (‘anomalous jaguar’) and CF-0023 (‘Peruvian tiger’).


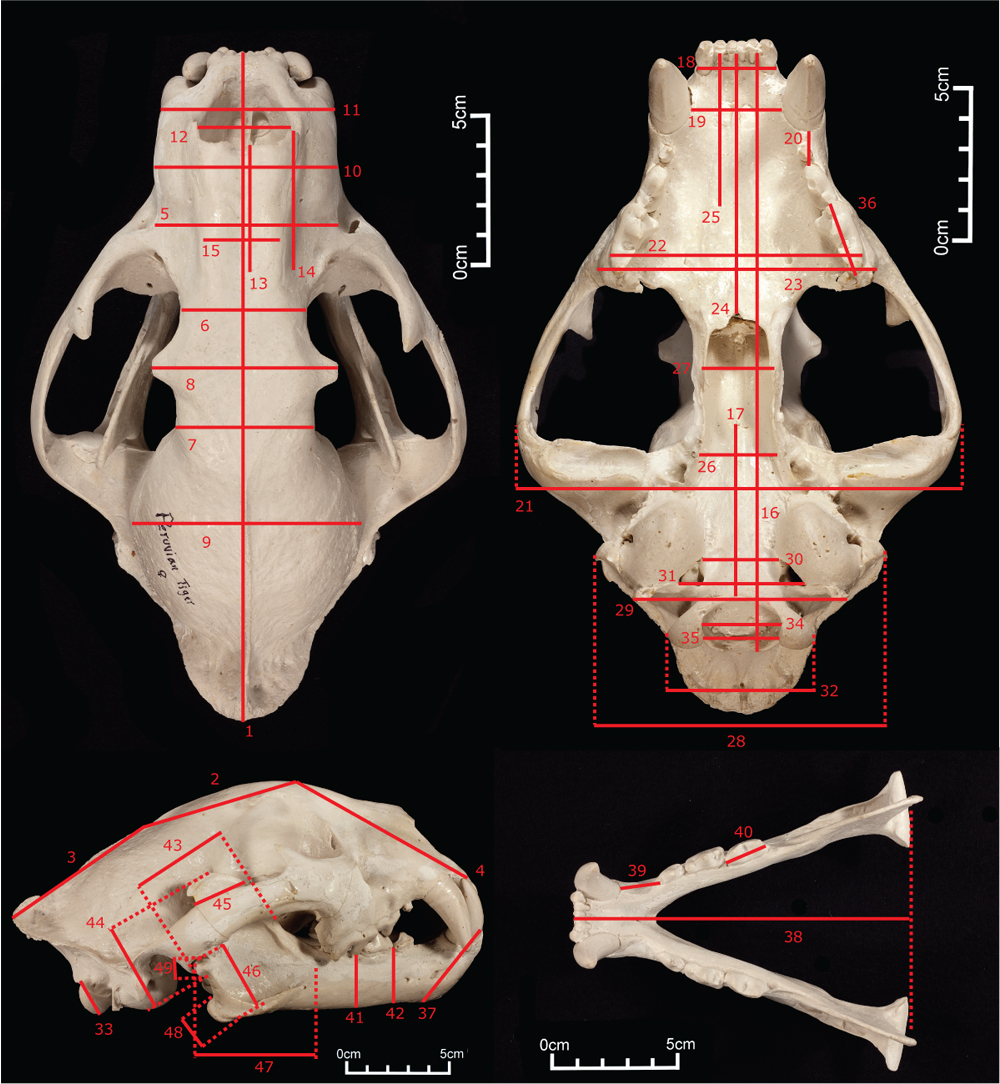


Craniomandibular measurements recorded from both skulls. The 36 cranial and 13 mandibular measurements are illustrated on the replica skull and mandible of the ‘Peruvian tiger’ (CF-0023). Values were recorded using a digital caliper. Numbers as in Table 1.

TABLE 1. Cranial and mandibular measurements of the two Peruvian cats in mm. *, mean value of left and right measurements.

Variable ‘Anomalous jaguar’ ‘Peruvian tiger’

1. *L*_SkT_ 250 218
2. *L*_Fr_ 76 67
3. *L*_Pa-Oc_ 107 87
4. *L*_face_ 104 88
5. *W*_iof_ 64 58
6. *W*_o_ 44 39
7. *W*_POC_ 43 43
8. *W*_POP_ 61 57
9. *W*_BC_ 76 75
10. *W*_sn_ 68 59
11. *W*_C1s_ 70 58
12. *W*_NA_ 31 28
13. *L*_N_ 55 44
14. *L*_NT_ 65 54
15. *W*_MFS_ 23 23
16. *L*_CdBs_ 223 193
17. *L*_BsCr_ 68 59
18. *W*_IC1s_ 36 31
19. *W*_I3s-I3s_ 30 25
20. *L*_C1sP3s_ 11 11.5
21. *W*_sk_ 162 144
22. *W*_P4s-P4s_ 88 80
23. *W*_pal_ 97 86
24. *L*_pal_ 104 91
25. *L*_PMMX_ 62 52
26. *W*_PN_ 20 20
27. *W*_PN.ant_ 22 21
28. *W*_MP_ 101 92
29. *W*_PocP_ 77 68
30. *d*_AB.ant_ 25 25
31. *d*_AB.post_ 45 39
32. *W*_OC_ 48 47
33. *H*_OC_ 21 18
34. *W*_FM_ 25 26
35. *W*_FMV_ 26 23
36. *L*_P4s_^*^ 28.7 25.2
37. *L*_Sym_ 55 48
38. *L*_Mand_^*^ 159 141
39. *L*_Diast_^*^ 13.5 16
40. *L*_m1_^*^ 20.95 18.35
41. *D*_MdM1_^*^ 33 28
42. *D*_MdP4_^*^ 31.5 26.5
43. *R*_T1_^*^ 55 44.5
44. *R*_T2_^*^ 55 47.5
45. *L*_CorProc_^*^ 32.5 28.5
46. *R*_M1_^*^ 34.5 32
47. *R*_M2_^*^ 71.5 66
48. *D*_AngProc_^*^ 14 14
49. *D*_Cond_^*^ 12.5 12

**Definitions of cranial and mandibular morphometric variables**

1. *L*_SKT_, greatest skull length
2. *L*_Fr_, frontal length
3. *L*_Pa-Oc_, parietal-occipital length
4. *L*_face_, facial length
5. *W*_iof_, snout width at the infraorbital foramen
6. *W*_o_, interorbital width
7. *W*_POC_, postorbital constriction width
8. *W*_POP_, postorbital process width
9. *W*_BC_, braincase width
10. *W*_sn_, snout width at approximate midsnout
11. *W*_C1s_, snout width at canines
12. *W*_NA_, nasal aperture width
13. *L*_N_, nasal length
14. *L*_NT_, total nasal length
15. *W*_MFS_, nasal width at maxillo-frontal suture
16. *L*_CdBs_, condylo-basal length
17. *L*_BsCr_, basicranial length
18. *W*_IC1s_, intercanine distance
19. *W*_I3s-I3s_, incisor arc width
20. *L*_C1sP3s_, C1-P3 distance
21. *W*_sk_, skull width
22. *W*_P4s-P4s_, palatal width at P4
23. *W*_pal_, palatal width posterior to P4
24. *L*_pal_, palatal length
25. *L*_PMMX_, premaxillary-maxillary length along palatal midline
26. *W*_PN_, postnarial passage width
27. *W*_PN.ant_, postnarial passage width at the palatal margin
28. *W*_MP_, mastoid process width
29. *W*_PocP_, paroccipital process width
30. *d*_AB.ant_, auditory bullae distance at the anterior margin
31. *d*_AB.post_, auditory bullae distance at posterior margin
32. *W*_OC_, occipital condylar width
33. *H*_OC_, occipital condylar height
34. *W*_FM_, foramen magnum width
35. *W*_FMV_, foramen magnum width the ventral margin
36. *L*_P4s_, P4 length
37. *L*_Sym_, mandibular symphysis length
38. *L*_Mand_, mandibular length
39. *L*_Diast_, diastema length
40. *L*_m1_, m1 length
41. *D*_MdM1_, mandibular depth at m1
42. *D*_MdP4_, mandibular depth at p4
43. *R*_T1_, temporal muscle lever arm1
44. *R*_T2_, temporal muscle lever arm2
45. *L*_CorProc_, antero-posterior coronoid process length
46. *R*_M1_, masseter muscle lever arm1
47. *R*_M2_, masseter muscle lever arm2
48. *D*_AngProc_, angular process depth
49. *D*_Cond_, condylar depth
